# Engineered tissue vascularization and engraftment depends on host model

**DOI:** 10.1038/s41598-022-23895-2

**Published:** 2023-02-03

**Authors:** Eileen L. Brady, Olivia Prado, Fredrik Johansson, Shannon N. Mitchell, Amy M. Martinson, Elaheh Karbassi, Hans Reinecke, Charles E. Murry, Jennifer Davis, Kelly R. Stevens

**Affiliations:** 1 Department of Bioengineering, University of Washington, Seattle, WA 98195 USA; 2 Institute for Stem Cell and Regenerative Medicine, Seattle, WA 98195 USA; 3 Department of Laboratory Medicine and Pathology, University of Washington, Seattle, WA 98195 USA; 4 Center for Cardiovascular Biology, University of Washington, Seattle, WA 98195 USA; 5 Brotman Baty Institute, Seattle, WA 98195 USA

**Keywords:** Biomaterials, Cell delivery, Stem-cell biotechnology, Tissue engineering

## Abstract

Developing vascular networks that integrate with the host circulation and support cells engrafted within engineered tissues remains a key challenge in tissue engineering. Most previous work in this field has focused on developing new methods to build human vascular networks within engineered tissues prior to their implant in vivo, with substantively less attention paid to the role of the host in tissue vascularization and engraftment. Here, we assessed the role that different host animal models and anatomic implant locations play in vascularization and cardiomyocyte survival within engineered tissues. We found major differences in the formation of graft-derived blood vessels and survival of cardiomyocytes after implantation of identical tissues in immunodeficient athymic nude mice *versus* rats. Athymic mice supported robust guided vascularization of human microvessels carrying host blood but relatively sparse cardiac grafts within engineered tissues, regardless of implant site. Conversely, athymic rats produced substantive inflammatory changes that degraded grafts (abdomen) or disrupted vascular patterning (heart). Despite disrupted vascular patterning, athymic rats supported > 3-fold larger human cardiomyocyte grafts compared to athymic mice. This work demonstrates the critical importance of the host for vascularization and engraftment of engineered tissues, which has broad translational implications across regenerative medicine.

## Introduction

The need for transplantable organs far outpaces the number of available donor organs. As this discrepancy grows, an increasing number of patients on organ transplant waiting lists will die awaiting an organ^1^. Tissue engineering could address this problem by generating an alternative source of healthy human tissue for those unable to access donor organs. However, without vasculature, engineered tissues are limited in thickness by the diffusion limit of oxygen (100-200 μm)^2–4^. Thus, vascularization is required to replicate the tissue thickness and complexity of commonly transplanted organs such as liver, lung, or heart.

Major progress towards vascularizing engineered tissues arose from the discovery that endothelial cells have the capacity to self-assemble into microvessel-like networks in vivo. These early experiments found that when endothelial cells were encapsulated in natural (fibronectin/collagen)^5^ or synthetic (PLGA/PLLA)^6^ hydrogel scaffolds and implanted in immunodeficient mice, they formed tubular networks that connected (anastomosed) to host circulation and contained host blood. Later studies further showed that including both endothelial and stromal cells within engineered tissues enhanced the vascular self-assembly process, leading to the formation of perfused, vascular networks that were at least in part derived from the grafted cells^7,8^.

Despite this progress in leveraging cooperation between the graft and host for tissue vascularization, randomly self-assembled networks exhibited irregular vessel architecture, susceptibility to early thrombosis^9,10^, and a delay between implantation and perfusion during which sensitive cells are deprived of oxygen. To address these limitations, we and others have developed methods to exert additional control over vascular architecture within the engineered tissue^11,12^. We developed a method to pattern endothelial cells and collagen into arrays of parallel channels and then encapsulate the resultant “endothelial cords” within engineered tissues^11,12^. Upon implantation in athymic mice, these endothelial cords act as “railroad tracks” that guide the formation of chimeric host-graft vessels that became anastomosed with the host circulation. Importantly, inclusion of patterned vascular networks improved the survival of functional hepatocytes within engineered liver tissue over randomly organized networks^11^. Due to this early success, we then sought to extend our approach for guided vascularization for cardiac tissue engineering, by implanting engineered tissues with endothelial cords on athymic rat hearts^13^. We were surprised to find robust inflammation and no evidence of patterned vessels after tissue implantation^13^. These results drew our attention to the likely critical yet often overlooked role that factors such as host biology and anatomic location of the grafted tissue may play in affecting vascular integration and engraftment of engineered tissues. We hypothesized that different host models (here, animal models) and anatomic implant locations play a role in the vascularization of human engineered tissues upon their implantation in vivo.

Here, we test this hypothesis and report divergent responses in vascularization and cardiomyocyte engraftment of engineered tissues after their implantation in athymic mice *versus* athymic rats. These results suggest that guided vascularization and cardiomyocyte survival are supported by different host factors, which vary across animal models.

## Results

### Patches with endothelial “cords” retain patterning and anastomose with host circulation in the mouse abdomen and heart

We previously showed that patterning endothelial cells and collagen into cord-like structures within fibrin-based engineered tissues facilitates the guided formation of patterned, chimeric host-graft vessels upon their intraperitoneal (IP) implantation in the abdomen of athymic mice^11^. Yet paradoxically, we also showed recently that such engineered tissues are rapidly degraded and do not retain vessel patterning after 7 days of implantation on the heart of athymic rats^13^. We first hypothesized that differences in host response across anatomic locations of the implanted tissue, in this case between the heart and the abdomen, might explain these results.

**Figure 1:**
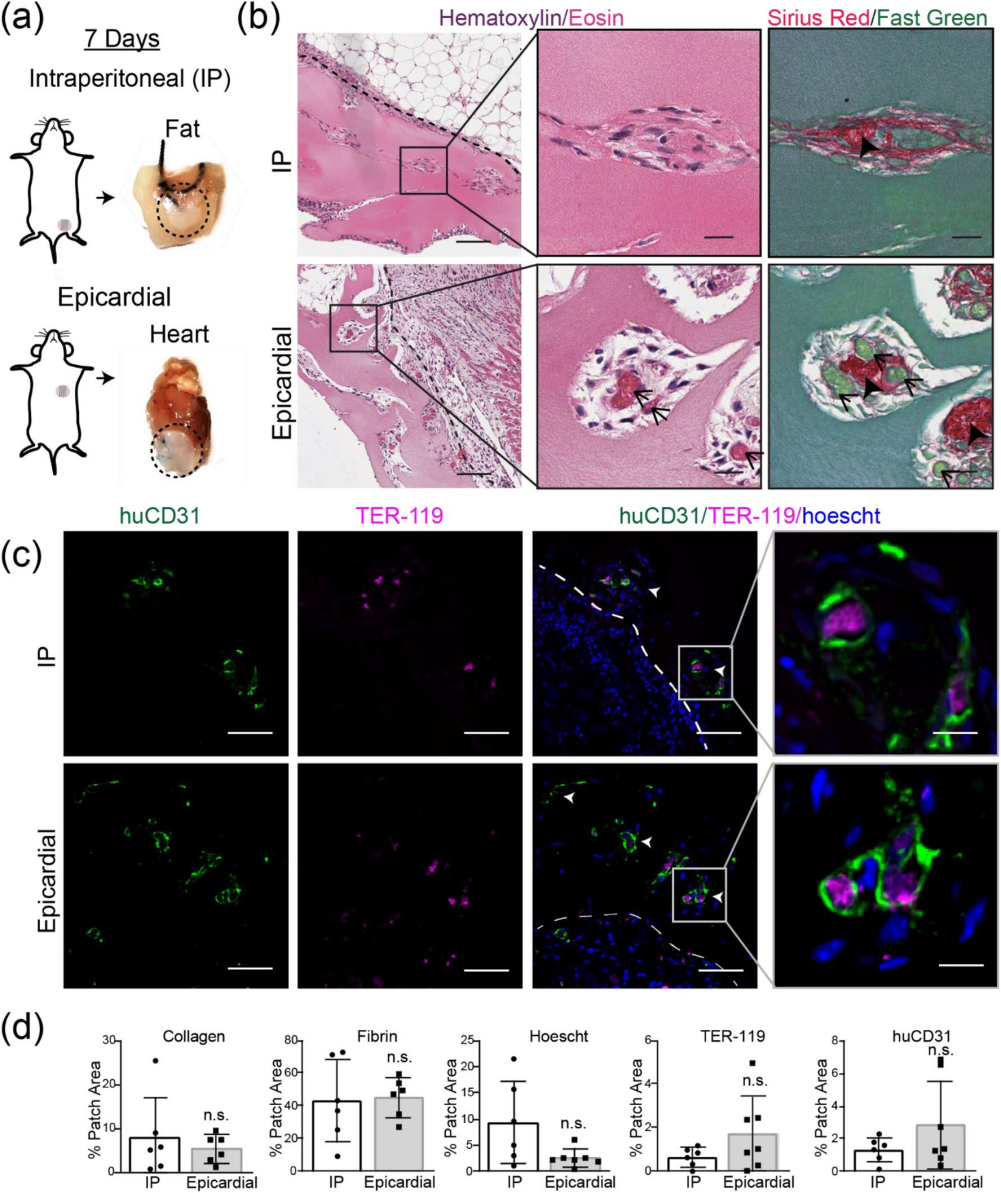
Guided vascularization occurs in Intra-peritoneal (IP) and epicardial locations in mice after 7 days of tissue implantation. (**a**) Explants from gonadal fat pad (IP) or epicardial (heart) locations have intact patches at 7 days (dotted lines). (**b**) H&E and Sirius Red/Fast Green stains of day 7 explants show cords-associated vessels in both IP and epicardial implants. Dashed line indicates host-graft boundary. Insets: open arrows indicate blood pools, closed arrows mark collagen cords. Scale bar = 100 μm (left). Inset scale bar = 20 μm. Immunostaining of day 7 explants (**c**) confirms regular clusters (white arrows) of graft-derived vessels (huCD31+) filled with mouse blood (TER-119+). Scale bar = 50 μm. Inset scale bar = 10 μm. (**d**) Quantification of staining by percent patch area shows no difference between groups in collagen, fibrin, nuclei, blood (TER-119), or huCD31. Each point represents an individual animal. Error bars reported as S.D. Collagen, fibrin n= 6 (IP) n = 6 (epicardial). Immunostaining (Hoeschst, TER-119, huCD31) n = 6 (IP), n = 7 (epicardial).

To methodically test this hypothesis here, we suspended endothelial cells (HUVECs) and stromal cells in collagen within a PDMS mold with parallel channels to form “endothelial cords”, then encapsulated these cords within fibrin to create engineered tissues, as we have done before^11^. We then randomly allocated these tissues into two groups and sutured the tissues from each group onto either the intraperitoneal (IP) gonadal fat pad or on the epicardial surface of the heart in athymic nude mice (Fig. 1a). These engineered tissues were explanted at either 3 or 7 days post implantation for histological analyses. Upon gross examination at explanted tissues, we identified intact engineered tissues with minimal adhesions at 3- and 7-day timepoints at each anatomic implant location (Fig. 1a). Furthermore, Hematoxylin and Eosin (H&E) and Sirius Red (SR) staining of sectioned explanted tissues revealed regularly spaced clusters of cells in a pattern reflecting cord cross-sectional geometry from both locations at 3 and 7 days (Fig. S1a, Fig. 1b). Within these clusters, these stains identified a collagen core surrounded by loosely organized cells at 3 days (Fig. S1b) that consolidated to form blood-containing lumens at the periphery of each cord by 7 days (Fig. 1b), similar to our previous results for tissues implanted IP in mice^11,12^. We next assessed inflammation by looking for nuclear infiltration and collagen deposition within the explanted engineered tissues. Whereas hematoxylin-positive nuclei were present at the grafted tissue boundary, nuclear infiltration did not extend into the graft, suggesting that the inflammatory response was contained at the host-graft interface. Furthermore, all explanted tissues had an intact Eosin-positive fibrin matrix without interstitial collagen deposition (Fig. 1b). Taken together, these results demonstrate a morphologic pattern of cord-associated lumen formation with minimal inflammation in both IP and epicardial implant locations in athymic nude mice.

To further explore the phenotype of cells in these structures, we performed immunostaining with a human specific anti-CD31 antibody (huCD31) and a mouse specific red blood cell marker (TER-119). At 3-days post implantation, huCD31+ cells remain primarily clustered at the periphery of the cords in the IP implants, with evidence of some rudimentary lumen formation more predominantly in the epicardial implants (Fig. S1b). By 7 days, the cords at both implant locations are lined with tracks of huCD31+ lumen that contain mouse red blood cells (RBCs) (Fig. 1c). Additionally, this staining confirmed that cords geometry was retained in patches from either implant location, with huCD31+ cells found almost exclusively in distinct clusters of lumens spaced approximately 50-100 µm apart (Fig. 1c, arrows). Quantification of collagen, fibrin, cellular nuclei (Hoescht), huCD31, and blood (TER-119) showed greater huCD31+ area in the epicardial group compared to IP implants at 3 days (Fig. S1c). However, by 7 days this had resolved and there were no significant differences in patch architecture, inflammation, or vascularization between implant locations (Fig. 1d). By 7 days in both groups, nearly 100% of cords were associated with TER-119 (RBC) containing huCD31+ lumens (Fig. S1d). Overall, our results demonstrate that guided vascularization^11^ occurs at both IP and epicardial (heart) implant locations in athymic nude mice.

### Cords-containing cardiac patches in mice develop patterned vessels with increased lumen size

**Figure 2:**
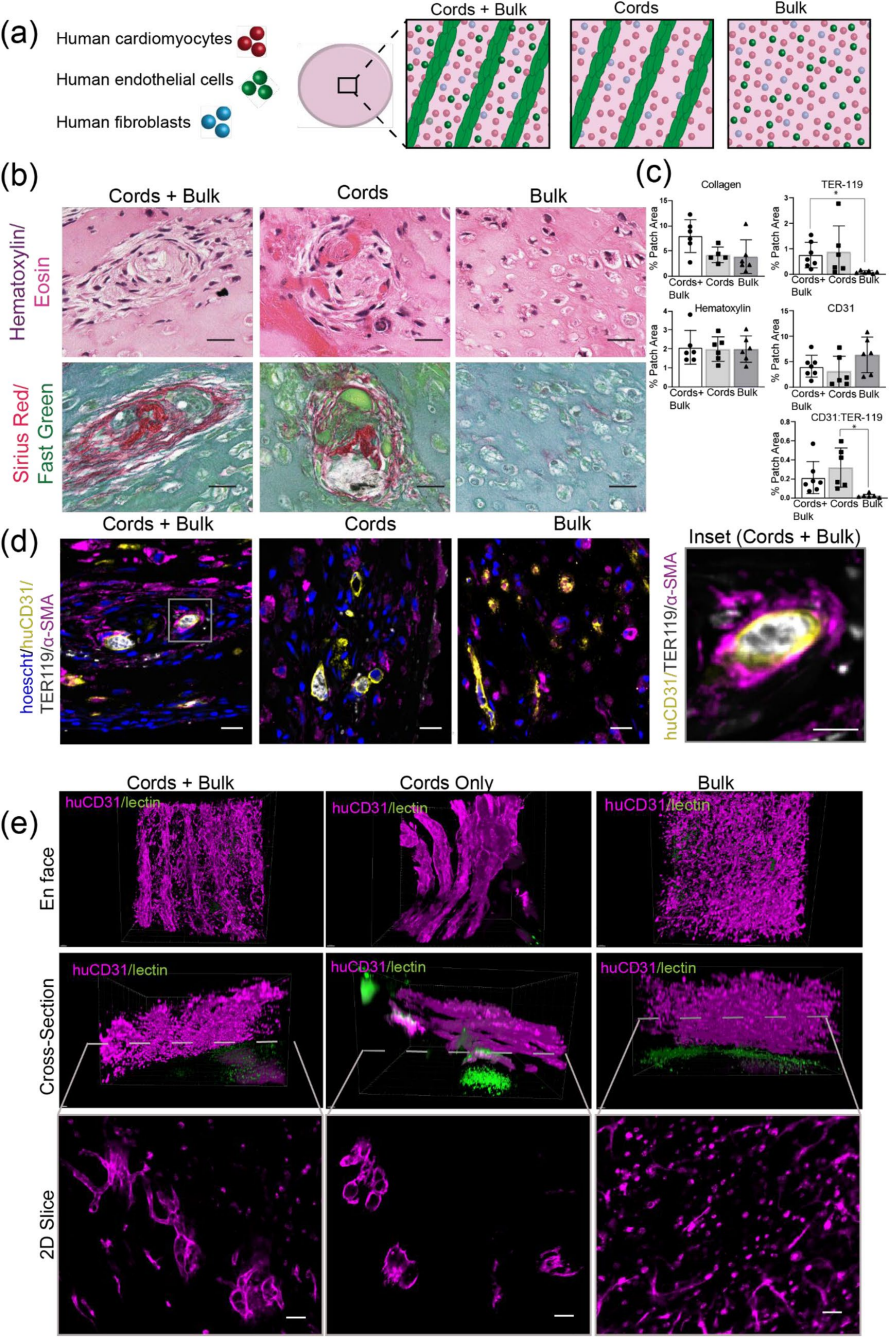
EC cords guide vascularization of engineered cardiac tissues in the athymic mouse heart. Patches were harvested at 7 days post-implantation. (**a**) Human cardiomyocytes, ECs, and stromal cells were used to create cardiac patches with different tissue geometries. (**b**) Hematoxylin & Eosin and Sirius Red demonstrate cords-associated blood pools in patches containing EC cords. Scale bar = 25 μm (**c**) Quantification of collagen, nuclei, huCD31, TER-119, and huCD31:TER-119 between patches with different EC configurations. Error bars report S.D. Collagen n = 6 (cords + bulk), n = 5 (cords), n = 6 (bulk). Hematoxylin n = 6 (cords + bulk), n = 6 (cords), n = 6 (bulk). Immunostaining (TER119/CD31) n = 7 (cords + bulk), n = 6 (cords), n = 6 (bulk). (**d**) Patterned huCD31+ vessels are filled with blood (TER-119) and recruit α-SMA pericytes. Scale bar = 20 μm. Inset scale bar = 10 μm. (**e**) Top: 3D visualization of huCD31 (magenta) and intravenously circulated fluorescent lectins (UEA-1 and LEL, green). Bottom: single 2D slice taken from z-stack at level indicated by dashed line. Scale bar = 50 μm.

After we had confirmed that guided vascularization occurs in the mouse heart, we next sought to apply this technique for cardiac tissue containing not only vascular cells, but also human pluripotent stem cell-derived cardiomyocytes (“cardiomyocytes”). We fabricated engineered tissues containing randomly seeded cardiomyocytes along with three endothelial cell patterning compositions: (1) patterned EC cords as well as randomly seeded ECs in the bulk (cords + bulk), (2) ECs in cords only (cords), (3) randomly seeded ECs in the bulk only (bulk) (Fig. 2a).

To compare vascularization between groups, we first used 2D histology to assess overall tissue structure and look for evidence of blood-vessels within the grafts after 7 days of engraftment, since our previous experiment (Fig. 1) had indicated that host-graft anastomosis occurs by this timepoint. Hematoxylin and Eosin and Sirius Red stains suggested guided vascularization had occurred, with blood pools associated with collagen cords in both cords + bulk and cords-only groups (Fig. 2b). Similar large blood consolidations were absent in patches lacking cords (Fig. 2b). Patches from all groups remained intact with minimal collagen deposition or excessive inflammation, with no significant differences between groups (Fig. 2c). To further compare graft-derived vessel morphology between groups, we co-stained for human endothelium (huCD31), mouse erythrocytes (TER-119), and the pericyte marker α-smooth muscle actin (α-SMA) (Fig. 2d). Compared to patches with only unpatterned ECs, we found that graft-derived endothelial cells in cords-containing groups more efficiently recruited blood (Fig. 2c). We then assessed for the presence of α-SMA+ pericytes, which are a marker of mature microvessels^14^. We found the cords-associated vessels often had nearby α-SMA+ cells (Fig. 2d). Additionally, some of these vessels had α-SMA+ cells partially or fully encircling the huCD31+ lumen, as would be expected in mature vessels (Fig. 2d, inset). The increased efficiency of blood recruitment and the presence of vessel associated α-SMA+ cells suggest that guided vascularization could support stable vessel formation in the mouse heart.

**Figure 3:**
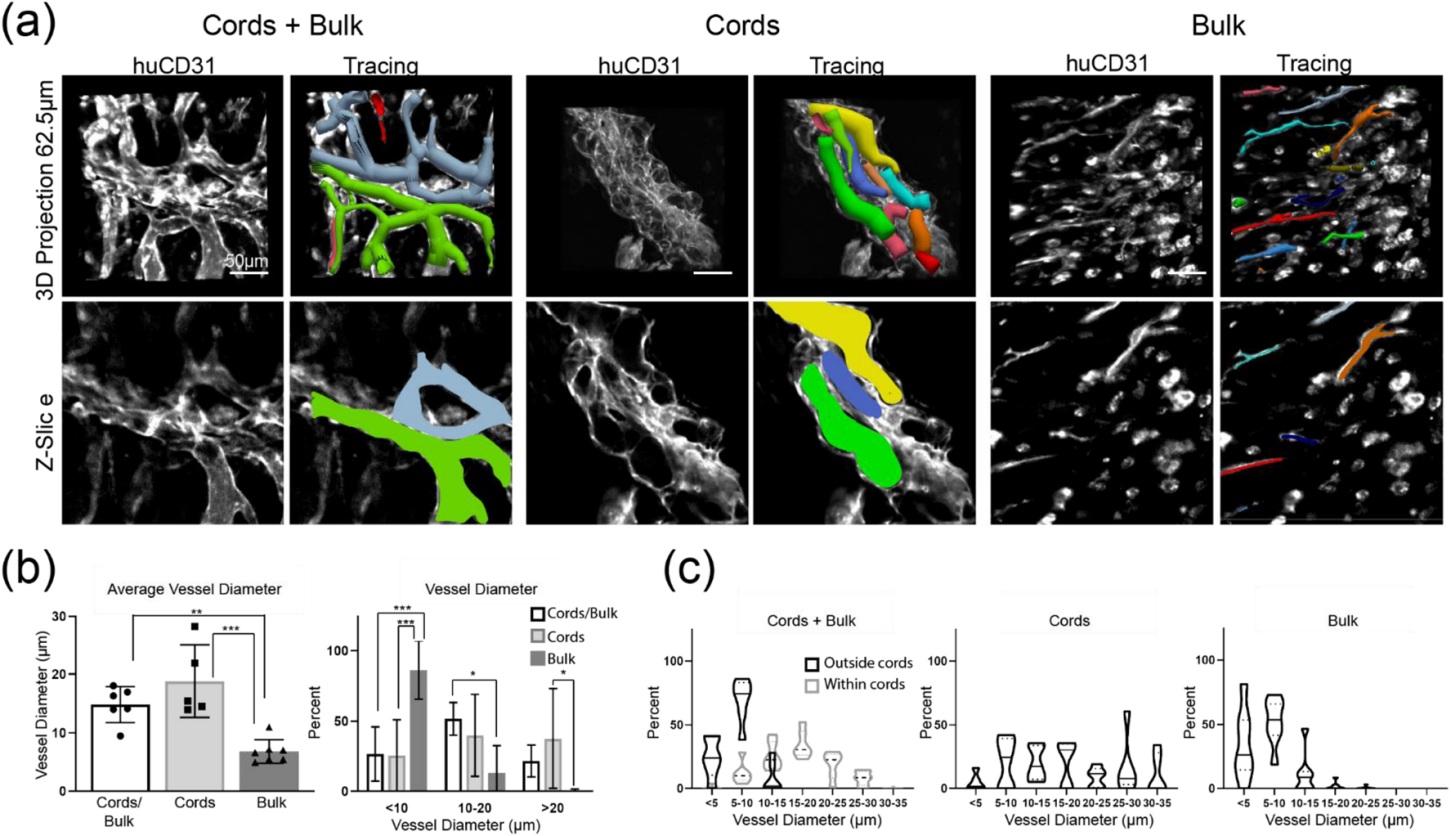
Vessel tracing and morphometric analysis. (**a**) Vesselucida software was used to trace all vessels within a 250μm^3^ ROI taken from the center of each patch. (**b**) Quantification of vessel diameter from traced vessels. Error bars indicate S.D. (**c**) Vessels found outside cords (black) are smaller than those found within cords (grey). n = 6 (cords + bulk), n = 5 (cords), n = 7 (bulk).

While the clustered pattern of graft-derived vessels in the 2D staining data suggested that vessels retained cords geometry in mice, we wanted to better understand three-dimensional (3D) engineered vascular geometry on the surface of the heart. To do this, we stained whole tissues for human endothelium (huCD31) and imaged the cleared samples with confocal microscopy. We also visualized perfused vessels using fluorescent lectin that had been introduced intravenously prior to harvest. In both cords-containing groups, we identified a parallel array of huCD31+ vessels (Fig. 2e). Cross-sections of these cords revealed hollow 20–40 μm lumens forming “trunks” aligned with the axis of each “cord”, and smaller vessels branching orthogonally from these larger trunks. No patterning was evident in the bulk-only patches. Instead, the bulk-only tissues had small vessel-like structures scattered throughout and many huCD31+ cells remained as isolated cells not incorporated into vessels (Fig. 2e). The group containing both cords and HUVECs in the bulk had features of both the cords and bulk-only groups, with larger vessels in the cords and smaller vessels and unincorporated huCD31+ cells throughout. To quantify the observed differences in vessel size between cords-associated vessels and those derived from randomly seeded HUVECs, we used Vesselucida software to generate tracings of vessels from each engineered tissue (Fig. 3a, Suppl Videos 1, 2, 3). Analysis of these tracings showed that vessels in the bulk-only group were significantly smaller than those in the cords-containing groups, with an average of 86% of vessels in bulk-only patches having a diameter < 10 μm and less than 1% having diameter > 20 μm (Fig. 3b). The cords-associated vessels were significantly larger, with an average diameter close to 20 μm (Fig. 3b). In the tissues with both cords and bulk HUVECs, vessels in the cords (black) more closely matched the cords-only group, while vessels in the bulk (gray) were similar in size to those in the bulk-only cohort (Fig. 3c). While 2D immunostaining identified blood within graft-derived lumens, indicating anastomosis with the host circulation, we very rarely observed lectin + vessels in the patches in any group (Fig. 2e), suggesting that flow in patches was too slow to be detected in the lectin circulation time. Taken together, these data indicate that in the athymic mouse heart EC cords guide the formation of patterned blood-filled vessels with larger lumen sizes compared to vessels assembled from homogeneously seeded cells, albeit with circulation that remains lower than that of the host coronary vasculature. Thus, guiding vascularization with EC cords may be a viable strategy for cardiac tissue engineering applications in an appropriate host setting.

### Animal model differences in response to cords-containing human cardiac patches

From our studies of cords-containing cardiac tissues in mice, we saw that vascular patterning was retained, and furthermore that endothelial cords influence the organization and size of vessels formed in vivo. Yet we were surprised to find that despite vessel formation and anastomosis in vivo, the human cardiomyocyte grafts remained rather small and dispersed across all conditions (Fig. S2). We found this particularly interesting since we and others have observed substantive cardiac grafts previously in other settings, such as in the athymic nude rat. Thus, we wanted to further explore the host response to grafts in athymic nude mice *versus* rats.

As a first step, we performed an “implant location” study in athymic nude rats, as we had done in athymic nude mice (Fig. 1). We implanted identical fibrin patches containing endothelial cords either in the abdomen (IP space) or the epicardial surface of athymic rats. Upon retrieval of the tissues from the IP location, we were able to identify the location of the implant by visualizing the suture but could not identify any of the engineered tissues in any of the animals, either by gross observation or upon histologic examination through the plane of the suture (Fig. S3). Conversely, all epicardial implants were identified grossly at the time of explant and further histologically processed. Histological staining of the epicardial explants with Hematoxylin and Eosin and Sirius Red revealed an inflammatory reaction characterized by nuclear infiltration, collagen deposition, and minimal remaining fibrin matrix (Fig. S4a), consistent with our previous findings^13^. Further immunostaining revealed numerous CD68+ macrophages throughout the tissue and concentrated around the perimeter of the remaining fibrin (Fig. S4b), as well as microvessels lined with huCD31+ graft-derived endothelial cells scattered throughout the graft (Fig. S4c). Further 3D huCD31 staining, clearing, and imaging of whole tissues (Fig. S4d) demonstrated the presence of human vessel structures with diameters in the range of 5–10 μm, with minimal architectural patterning (Fig. S4d). Thus, athymic rats appeared to produce a robust inflammatory response that seemingly degrades engineered tissues in the IP space and disrupts vessel patterning in those implanted on the epicardial surface of the heart^13^.

**Figure 4:**
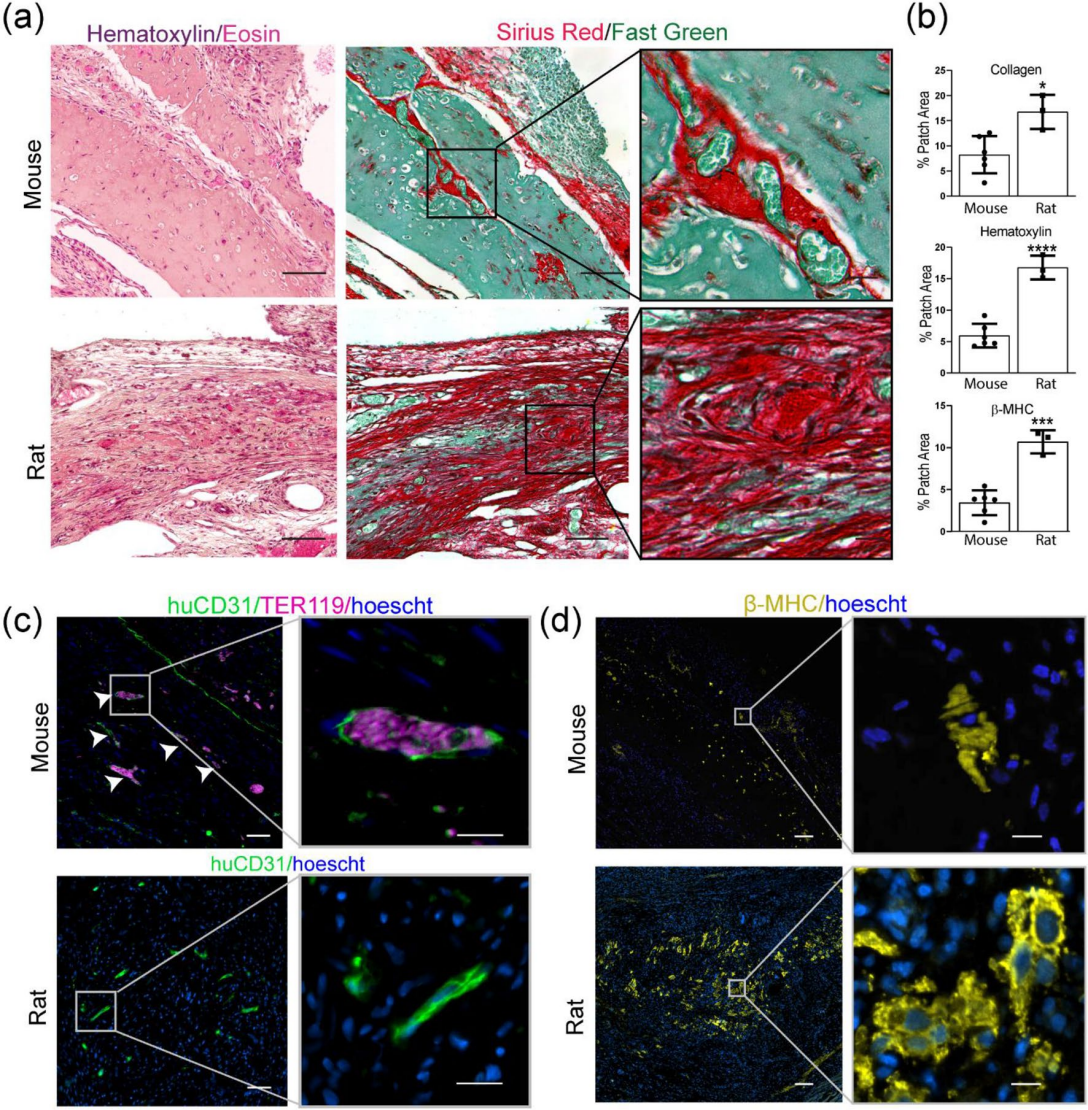
Cords-containing cardiac patch induces patterned vasculature in mice, but robust inflammation in rat at 10 days post-implantation. (**a**) H&E and Sirius Red/Fast Green show hematoxylin-positive nuclear infiltrate and SR+ collagen in patches implanted in rats, but minimal inflammation in mice. Patches in mice have blood pools associated with cords (inset). Scale bar = 100 μm. Inset scale bar = 20 μm. (**b**) Quantification of collagen, nuclei, and human myocardium as percent of patch area in rats and mice. Error bars report S.D. n = 6 (mice) n = 3 (rats). (**c**) In mice, huCD31 staining reveals cords-associated clusters of graft-derived vessels filled with TER-119 + blood (white arrows indicate cords). huCD31 staining in rats fails to recapitulate cords patterning. Scale bar = 50 μm. Inset scale bar = 20 μm (**d**) Human cardiac graft size is larger in rats than mice. Scale bar = 100 μm. Inset scale bar = 10 μm.

We next directly compared engraftment of engineered cardiac tissues on the epicardial surface of athymic mouse or rat hearts. We fabricated cardiac tissues containing endothelial cells in both cords and the tissue bulk, as well as human cardiomyocytes (Suppl Fig. S6 and Suppl Video 4). These tissues were then distributed for implant on the hearts of athymic rats or mice. Upon retrieval of the tissues 10 days post-implantation, we found that the explanted tissues from mice had evidence of guided vascularization, with Hematoxylin & Eosin and Sirius Red/Fast Green staining showing large blood-filled lumens in a linear pattern reminiscent of cord cross-sections and Sirius Red staining confirming these were associated with collagen cords, similar to our previous results (Fig. 4a). In contrast, tissues recovered from rats had no evidence of cords-associated blood consolidations (Fig. 4a). Instead, most of the tissue was replaced by a collagen matrix in rats, with > 3-fold more collagen found in tissues in rats compared to those in in mice (Fig. 4b). While little fibrin remained in rat explants, the tissues recovered from mice contained primarily Eosin-stained fibrin, with some collagen present at the graft-host boundary and within the cords (Fig. 4a). We also noted substantively more Hematoxylin-positive nuclei, indicative of an inflammatory response, in the tissues recovered from athymic rats compared to mice (Fig. 4a,b).

Finally, we performed immunostaining to identify human graft-derived endothelial cells and cardiomyocytes in explanted tissues from the two animal models. Immunostaining revealed that cords-containing patches in mice had distinct clusters of huCD31+ lumens that contained TER-119+ mouse blood, whereas the tissues explanted from rats had no discernible patterning of graft-derived cells (Fig. 4c). However, immunostaining for rodent specific endothelium in explanted tissues from both animal models showed no significant differences in host-derived vasculature (Fig. S7). To further assess human cardiomyocyte graft size within explanted tissues, we stained for human myocardium with β-myosin heavy chain (β-MHC) (Fig. 4d). Interestingly, grafts explanted from athymic rats were > 3-fold larger grafts explanted from mice (Fig. 4b). Morphologically, β-MHC cells in the tissue grafts explanted from rats were closely packed with larger cells that sometimes appeared elongated, while cells in the grafts in mice were isolated and small with a rounder, more punctate appearance (Fig. 4d, inset). Thus, while vascular patterning was retained in mice but disrupted in rats, cardiomyocyte engraftment as measured by overall graft size was paradoxically superior in rats despite apparent inflammation. These surprising results indicate a major variable that has been previously overlooked by our field, by demonstrating that identical tissues, when implanted in different host model systems, will yield vastly different engraftment results.

## Discussion

We previously demonstrated that endothelial cell “cords” encapsulated in a fibrin-based tissue guide the formation of host-perfused vessels along the axis of each cord in athymic mice^11^. However, when we attempted to replicate this strategy in a cardiac patch implanted in athymic rats, the cords led to only transient vessel patterning. In addition to loss of vessel patterning, we also observed a robust immune response leading to patch degradation and collagen deposition. Here, we meticulously assessed how host variables—anatomic implant location and animal model—differentially support guided vascularization and cardiac engraftment.

We first postulated that anatomic implant location may be the major variable at play in our studies, as others have reported a particularly robust immune response to biomaterials implanted in the epicardial environment compared to other anatomic locations^16,17^. For example, Kellar et. al implanted collagen discs epicardially in mice and found increased leukocyte recruitment, inflammatory cytokine expression, and matrix degradation compared with subcutaneous implants^17^. Similarly, ePTFE discs implanted epicardially in rats showed increased immune cell density compared with either subcutaneous or abdominal implants^16^. Interestingly, we found no differences in tissue morphology or vascularization between engineered tissues with EC “cords” implanted in either the IP space or on the heart in nude mice. We speculate that our failure to detect a difference between implant locations here could be explained by strain differences in immune response of athymic nude mice, as previous studies reporting increased inflammation in in epicardial implants have used immune-competent strains such as C56Bl/6 mice. The discrepancies between such studies thus may once again highlight the role for host factors and model systems as playing a role in tissue engineering outcomes.

Having found that athymic nude mice support guided vascularization in epicardial implants, we next sought to apply this strategy in the context of cardiac tissue engineering. By comparing engineered cardiac tissues with and without EC cords, we found that patches with EC cords more efficiently recruit host blood compared with unpatterned controls and that patterning endothelial cells in this way may allow control over vessel size in vivo. Additionally, a portion of cord-derived blood vessels were associated with α-SMA+ pericytes, a marker of mature vessel phenotype. We speculate that these pericytes are derived from the mouse mesenchymal cell line (CH310t1/2) incorporated within the cords, as others have shown can occur within a pro-vasculogenic environment^15^.These results represent a step forward towards replicating the hierarchy of natural vascular networks in permissive host settings in vivo, as guided vascularization led to the formation of larger vessel “trunks” with lumen diameters of 20–40 μm and smaller 5–15 μm vessels forming “branches”.

While we had confirmed that guided vascularization can be used successfully for cardiac tissue engineering applications in an appropriate host (athymic mouse), we still had not explained why this technique failed to perform similarly in the athymic rat heart^13^. To answer this question, we directly compared human cardiac tissues with EC cords implanted epicardially in either athymic mice or athymic rats. We found that guided vascularization occurred only in athymic mice, whereas tissues in athymic rats instead exhibited signs of inflammation. Despite this failure of guided vascularization, rats supported larger human cardiac grafts compared to patches implanted in mice, indicating that vascularization and cardiomyocyte engraftment require different host environments. These data suggest that in the future, host factors could potentially be leveraged to improve engraftment of vascular or parenchymal cells such as cardiomyocytes.

Based on the dramatic difference we observed in inflammatory response between these models, we speculated that a heightened innate immune response in the athymic rat heart may promote cardiomyocyte engraftment while interfering with vascularization. Athymic nude mice and athymic nude rats both lack mature T lymphocytes, but retain other immune cell types including macrophages, neutrophils, dendritic cells, natural killer (NK) cells, and B cells. Despite their apparently similar immune systems, differences in immune function between these models has been reported^18,19^. For example, while NK cell activity is elevated in both athymic rodent models, athymic rats have very high NK activity in the peritoneal cavity, whereas athymic mice have relatively low peritoneal NK activity^20^. It is possible that this animal model difference could explain our finding that abdominal cords implants remained intact in the mouse abdomen but were obliterated in the rat. In addition to heightened NK activity, enhanced macrophage activity has been reported in both athymic mice^21^ and athymic rats^19^. Mounting evidence points to the critical role of innate immunity in cardiac regenerative medicine^22,23^. In fact, recent groundbreaking work has suggested that the success of cellular therapies in the heart is primarily explained by the macrophage-predominant immune response induced by these therapies^23^. We thus speculate that a heightened innate immune response in athymic nude rats compared to athymic nude mice contributed to superior cardiomyocyte engraftment while interfering with vessel patterning^23^.

Taken together, our results suggest that the tissue engineering and cellular therapy fields would benefit from paying more careful attention to understanding and tuning host environment to achieve clinical success and ensure reproducibility. A challenge across biomedical research is the widespread replication crisis, which wastes limited resources, erodes public confidence in science, and impedes progress towards clinical translation^24^. Within tissue engineering and regenerative medicine, the neglected role of host biology may contribute to the high rate of pre-clinical failure and irreproducible findings^25^. This is likely particularly important in the pre-clinical development process, as new technologies are typically tested in increasingly larger animal models prior to human clinical trials. We suggest that increased attention to host biology could improve reproducibility and accelerate clinical translation of regenerative therapies, and potentially even be deployed to greatly enhance the efficacy of regenerative engineering strategies.

## Methods

### Cell culture and fabrication of cords patches

Human Umbilical Vein Endothelial Cells (Lonza, P4–P6) were cultured in EGM-2 media (Lonza) on tissue culture treated poly(styrene). Mouse mesenchymal cells CH310t1/2 (ATCC, P5–P7) were cultured in low glucose Dulbecco’s Modified Eagle’s Medium (DMEM) supplemented with 10% v/v fetal bovine serum (FBS, BioWest). Normal Human Dermal Fibroblasts (Lonza, P5–P7) were cultured in DMEM supplemented with 10% FBS. Media was replaced every 48 h and cells were passaged or used for experiments at 80% confluence. Patches containing endothelial cords were fabricated as has been previously described^11^. Briefly, HUVECs and CH310t1/2 cells were resuspended in a 2.5% collagen solution and introduced into a PDMS mold containing channels 150 μm wide. Excess collagen was aspirated from the mold leaving the cell/collagen mixture only in the channels. Collagen polymerization was achieved by incubating the filled molds at 37 °C for 8 min. Following polymerization, molds were incubated with EGM-2 media at 37 °C for 3 h to allow cord formation. Cords were then removed from the mold by encapsulating in a 1:1 mixture of 20 mg/mL fibrinogen (Sigma) and 2.5U/mL thrombin (Sigma) that was allowed to polymerize. Last, a 6 mm biopsy punch was used to punch out patches for implantation. Patches were then kept on ice floating in sterile EBM-2 (no supplements) until implantation.

For all cardiac patches, hPSC-CMs were prepared for implantation with heat-shock^26^ and a pro-survival cocktail^27^. 24 h prior to implantation, cardiomyocytes were changed to RPMI/BSA/Ascorbic acid that had been pre-warmed to 42 °C and plates were incubated for 30 min at 42 °C. After 30-min heat shock, media was changed to RBA supplemented with 100 ng/mL IGF-1 (Peprotech) and 200 nM Cyclosporine A (Wako). The next day, 1 h prior to trypsinization, media was changed to RBA supplemented with pro-survival cocktail (100 ng/mL IGF-1, 200 nM Cyclosporine A, 10 μM ZVAD-FMK (Calbiochem), 50 nM TAT-BH4 (Calbiochem), 50 μM pinacidil monohydrate (Sigma)). Immediately before the cord encapsulation step, 2 × 10^6^ hCMs and 5 × 10^5^ NHDFs were collected and resuspended in 300 μl fibrin for each cords mold. For groups with HUVECs in the bulk, 1 × 10^6^ HUVECs per mold were also mixed with hCMs and NHDFs. The pooled cells were then resuspended in 20 mg/mL fibrinogen and mixed thoroughly in a 1:1 ratio with thrombin to encapsulate cells in the bulk surrounding the cords. After fabrication, patches containing cardiomyocytes were kept on ice floating in sterile EBM-2 supplemented with pro-survival cocktail supplements described above. Please see Table 1 below for detailed description of cells in the bulk gel for each experiment.

**Table 1:** Cell types contained in patches for each experiment.

	Cords	HUVECs (bulk)	hPSC-CMs (bulk)	NHDFs (bulk)
Mouse (Fig. 1)	Yes	No	No	No
Mouse cords (Figs. 2, 3)	Yes	No	6.67 × 10^6^/mL	1.67 × 10^6^/mL
Mouse bulk (Figs. 2, 3)	No	3.33 × 10^6^/mL	6.67 × 10^6^/mL	1.67 × 10^6^/mL
Mouse cords + bulk (Figs. 2,3)	Yes	3.33 × 10^6^/mL	6.67 × 10^6^/mL	1.67 × 10^6^/mL
Mouse (Fig. 4)	Yes	3.33 × 10^6^/mL	6.67 × 10^6^/mL	1.67 × 10^6^/mL
Rat (Fig. 4)	Yes	3.33 × 10^6^/mL	6.67 × 10^6^/mL	1.67 × 10^6^/mL
Rat (Figs. S3 and S4)	Yes	No	No	No

### Maintenance and differentiation of pluripotent stem cells

Human PSCs (WTC-11 or H7, Coriell) were maintained with mTeSR-1 media (StemCell Technologies) on Matrigel (BD Biosciences). Directed differentiation to cardiomyocytes was achieved through modulation of the Wnt pathway using small molecules^28^. Briefly, on day 0 hPSCs were changed to mTesR with 1 μM Chiron 99021 (Tocris). After 24 h, media was changed to Roswell Park Memorial Institute (RPMI) 1640 media supplemented with B27 (minus insulin) containing 1 μM Chiron 99021. 48 h later, media was replaced with RPMI/B27(minus insulin) with 2 μM Wnt-C59 (Fisher). After 48 h, media was changed to the final cardio maintenance media, comprised of RPMI with B27 supplement containing insulin. Thereafter, media was replaced every 48 h. Between day 14 and 21, lactate selection with DMEM (no glucose) supplemented with 4 mM sodium lactate was performed for 72 h, after which cells were returned to cardio maintenance media (RPMI/B27)^29^. Cardiomyocytes were used for experiments either directly following differentiation or were frozen and thawed for later use. Frozen cardiomyocytes were thawed into RPMI with B27 supplement containing insulin, 5% FBS, and 10 μM ROCK inhibitor Y-27632 (StemCell Technologies) for 24 h and then returned to cardio maintenance media prior to use.

### In vivo implantation in mice and rats

For all mouse experiments, male athymic nude mice (Taconic, 8–10 weeks), were used. Male athymic nude Hsd:RH-Foxn1rnu rats (Envigo, 150–220 g) were used for rat studies. Sample size was determined prior to each experiment by reviewing the sample sizes required for statistical significance in similar published studies. For supra-epicardial patch placement, anesthesia was induced with either 5% isoflurane (rats) or ketamine/xylazine (130 mg/kg Ketamine, 8.8 mg/kg Xylazine, mice) preceding orotracheal intubation. Rats were maintained on 2% isoflurane and mice were supplemented with 0.5% isoflurane. The chest was opened via left lateral thoracotomy and the pericardium was removed to expose the heart. For rats, two 6 mm patches were affixed using 8-0 suture to the supra-epicardial surface. Due to their smaller heart size, mice received one 6 mm patch similarly affixed with 8-0 suture. The chest was closed, and animals were provided analgesia for the first two post-operative days via subcutaneous injection of slow releasing buprenorphine (1 mg/kg). For animals that received patches containing cardiomyocytes, daily injections of Cyclosporine A (5 mg/kg) were administered beginning one day prior to implantation to promote cardiomyocyte survival according to previously published pro-survival protocols^27^.

Animals receiving intra-peritoneal implants were induced with ketamine/xylazine (mice, 130 mg/kg Ketamine, 8.8 mg/kg Xylazine) or 5% isoflurane (rats) and a midline abdominal incision through the skin and peritoneum A single 6 mm patch was affixed with 5-0 suture to the gonadal fat pad. The incision was closed, and analgesia was provided for two days via slow releasing buprenorphine. Mice for IP experiments were harvested at day 3 or day 7 (Fig. 1 and Fig. S1) and rats for IP experiments were harvested at day 7 (Fig. S3). All animal procedures were approved by the University of Washington Institutional Animal Care and Use Committee (IACUC protocol #4388-02).

### Tissue harvesting, processing, and 2D histology

Animals were sacrificed at the pre-determined endpoint for each experiment (3, 7, or 10 days for mice; 10 days for rats). For mice receiving fluorescent lectins, 200 μl of a 1:1 mix of Dylight-649 conjugated Lycopersicon Esculentum (Tomato) Lectin (1 mg/mL, Vector Labs) and Dylight-649 conjugated Ulex Europaeus Lectin 1 (1 mg/mL, Vector Labs) was introduced via intravenous injection and allowed to circulate for 10 min prior to sacrifice. Tissues were fixed with 4% paraformaldehyde solution for 48 h and immediately used for whole tissue staining, clearing, and 3D imaging (see below), or progressively dehydrated for paraffin embedding. Paraffin blocks were sectioned at 5 μm with a microtome and sections collected on charged slides. Hematoxylin and Eosin (Harris hematoxylin and Eosin Y, Sigma) was used to visualize tissue morphometry. Collagen was identified with Picrosirius red (Direct Red 80, Sigma) using Fast Green (Sigma) as a counterstain. Prior to immunostaining, heat mediated antigen retrieval in pH 6.0 sodium citrate was performed. After blocking with 5% normal goat serum, primary antibodies were incubated at 4 degrees using antibodies against human CD31 (1:20, Dako), TER-119 (1:100, BD Biosciences), α-Smooth Muscle Actin (1:100, abcam), beta-myosin heavy chain (hybridoma supernatant, ATCC #CRL-2046, full strength), and 1:200 Dylight-649 conjugated Lycopersicon Esculentum (Tomato) Lectin (1 mg/mL, Vector Labs) followed by species-appropriate secondary antibodies conjugated to Alexa Fluor 488, AlexaFluor 555, or Alexa Fluor 647. Nuclei were visualized with 1:500 Hoescht 33342. Fluorescent images were acquired on a Nikon Eclipse Ti inverted microscope with either Photometrics CoolSNAP HQ2 camera for widefield images, or Yokogawa W1 spinning disk confocal head and Andor iXon Life EMCCD camera for confocal images. Images of H&E and Sirius Red stains were obtained from whole slide scans acquired with an Aperio ScanScope AT2 digital whole slide scanner or with a Nikon Eclipse Ti inverted microscope.

### Clearing and 3D imaging

Following fixation, whole tissues (gonadal fat pad or heart) were incubated for 6 h at 37 °C in a blocking solution (0.1 M Tris, 1% BSA, 1% Normal Donkey Serum, 0.3% triton x-100) followed by 24-h incubation with 1:100 anti-human CD31/PECAM-1 DyLight 550 conjugated antibody (Clone: JC/70A, Novus) at 37 °C on an orbital shaker. After staining, tissues were rendered optically transparent by clearing with the Clearing Enhanced 3D Microscopy (Ce3D) protocol at room temperature for 48 h^30^. Cleared tissues were imaged with a Nikon Eclipse Ti inverted microscope with Yokogawa W1 spinning disk confocal head and Andor iXon Life EMCCD camera. After samples were imaged, cleared tissues were incubated with PBS overnight to remove clearing agents. Tissues were then processed for paraffin embedding and 2D histology as described above. 3D datasets were visualized with IMARIS 3D visualization and analysis software (Oxford Instruments). Fibrin patches fixed pre-implantation were incubated in a blocking solution overnight followed by a 24-h incubation with 1:100 anti-human CD31/PECAM-1 DyLight 550 conjugated antibody (Clone: JC/70A, Novus) and 1:100 Recombinant Anti-Sarcomeric Alpha Actinin antibody (ab68167). Patches were then incubated in a species appropriate secondary antibody conjugated to AlexaFluor 647 and 1:500 Hoescht 33342 overnight and subsequently rendered optically transparent by clearing with the Clearing Enhanced 3D Microscopy (Ce3D) protocol at room temperature for 24 h^29^. Patches were imaged using a Leica SP8 confocal microscope.

### Quantitative tissue morphometry

For all analysis, patch area was defined as the region between the surface of the host tissue (fat or cardiac) and the edge of the patch. Quantification of graft size for all stains (β-MHC, TER-119, huCD31, Hoescht, H&E, and Sirius Red) was accomplished using FIJI by applying a uniform threshold across images and measuring positive area^31^. For 2D analyses, a slide from the mid-point of the patch taken selected for quantification and the entire patch was analyzed using a composite image constructed through image stitching using built in Nikon microscopy software.

For vessel tracing and morphometry, a 250 μm × 250 μm × 250 μm volume from the center of each patch was exported for analysis with the microvascular analysis suite Vesselucida (MBF biosciences). For the purposes of this analysis, “vessels” were defined as human CD31+ structures with continuous lumens. A semi-manual process was used for vessel tracing. First, small vessels were identified using the build in automatic tracing tool, which reliably identified vessels < 10 μm. Next, manual tracing was performed for all vessels not identified by automatic tracing. From these vessel tracings, the Vesselucida software generated data on vessel size for each network.

### Statistical analysis

Graphpad Prism software was used to complete all statistical analyses. For comparisons between two groups, an unpaired student t-test was performed. For analyses in which more than two groups were compared, a one-way ANOVA with Tukey’s HSD as post-hoc test was used for data with equal variance, or Welch’s Anova with Dunnett’s T3 multiple comparison test was applied for data with unequal variance. Error bars are reported as SD.

### Statement of compliance

All methods described were carried out in compliance with guidelines and regulations at the University of Washington. All animal procedures were approved by the University of Washington Institutional Animal Care and Use Committee (IACUC protocol #4388-02). Additionally, animal experiments were conducted and reported here in accordance with ARRIVE guidelines.

## Supplementary Information

Supplementary Figures.

Supplementary Video 1.

Supplementary Video 2.

Supplementary Video 3.

Supplementary Video 4.

## Data Availability

All the data needed to evaluate the conclusions made in this paper are present within the data presented in the paper and/or the Supplemental Materials. Additional data including raw/unprocessed files may be requested from the corresponding author (K.R.S.) at ksteve@uw.edu.
