# Supplementary material for: Engineered tissue vascularization and engraftment depends on host model
Source: Sci Rep. 2023 Feb 3;13:1973. doi: 10.1038/s41598-022-23895-2 (PMC9898562; doi:10.1038/s41598-022-23895-2)
Supplement: Supplementary file 1 — Supplementary Figures. [file 41598_2022_23895_MOESM1_ESM.docx]

Supplement

**
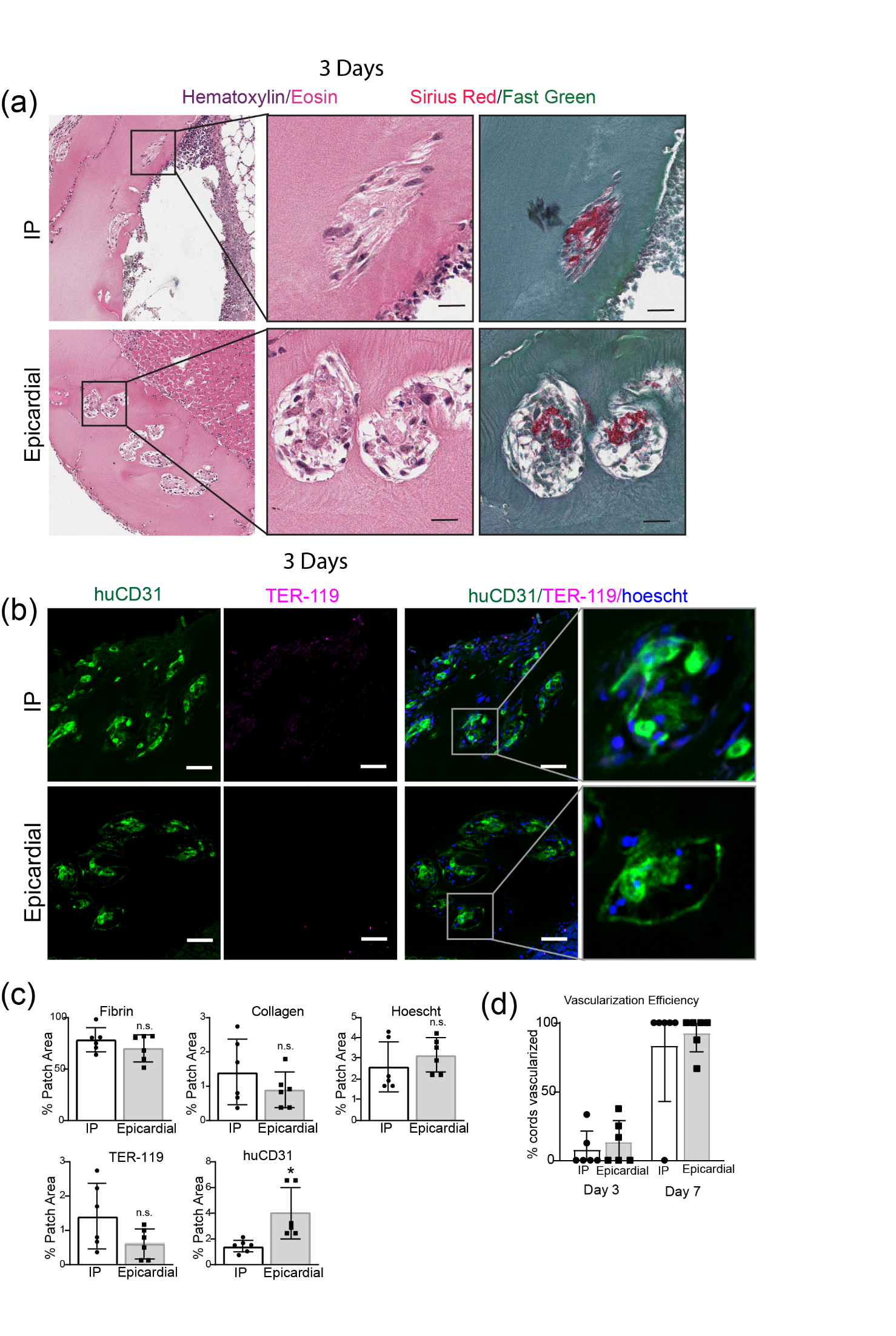
**

**Fig. S1.** 3-day epicardial and abdominal implants in mice. (a) H&E and Sirius Red/Fast Green stain of cords-containing fibrin patches 3 days post-implantation in mice. Scale bar = 200μm. Inset scale bar = 20μm. (b) Immunostaining of 3-day patches with huCD31 (green), TER-119 (magenta), and Hoescht (blue). Scale bar = 50μm. (c) Quantification of fibrin, collagen, Hoescht, TER-119, and huCD31 at day 3 reported as percent of patch area. Error bars show S.D. (d) Percentage of cords that are associated with blood-containing lumens (% cords vascularized). Error bars show S.D.


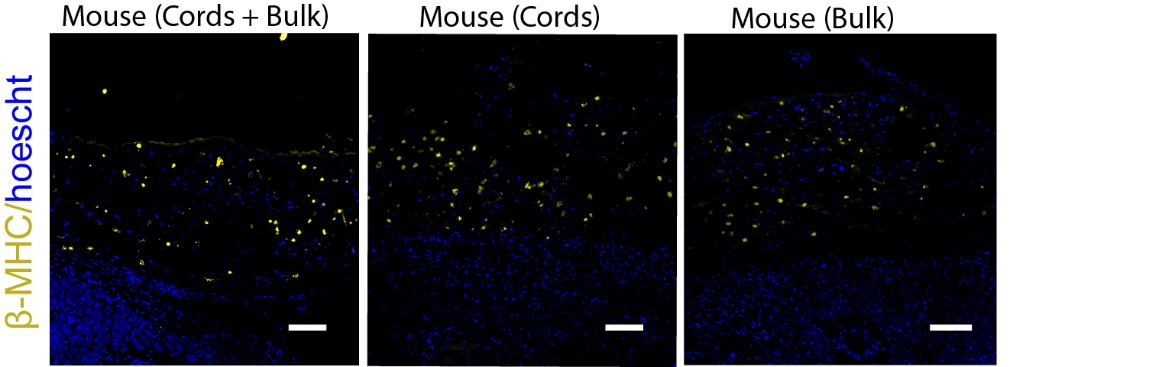


**Fig. S2.** β-MHC+ cardiac grafts in mice harvested at 7 days remain sparse despite guided vascularization. Scale bar = 100μm.

**
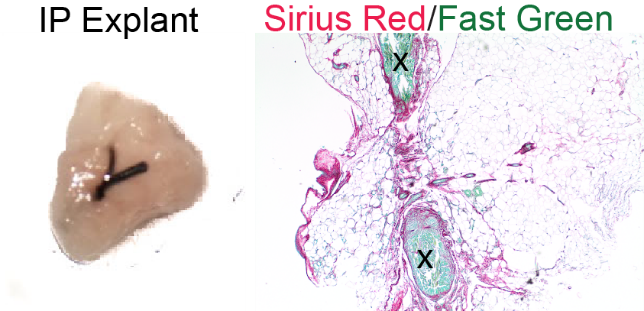
**

**Fig. S3.** Fibrin tissues containing EC cords are obliterated in the athymic rat IP space at 7 days. Left: explanted gonadal fat with intact suture but no visible patch. Right: Sirius Red/Fast Green through the middle of the suture (suture = x) fails to reveal remaining fibrin.

**
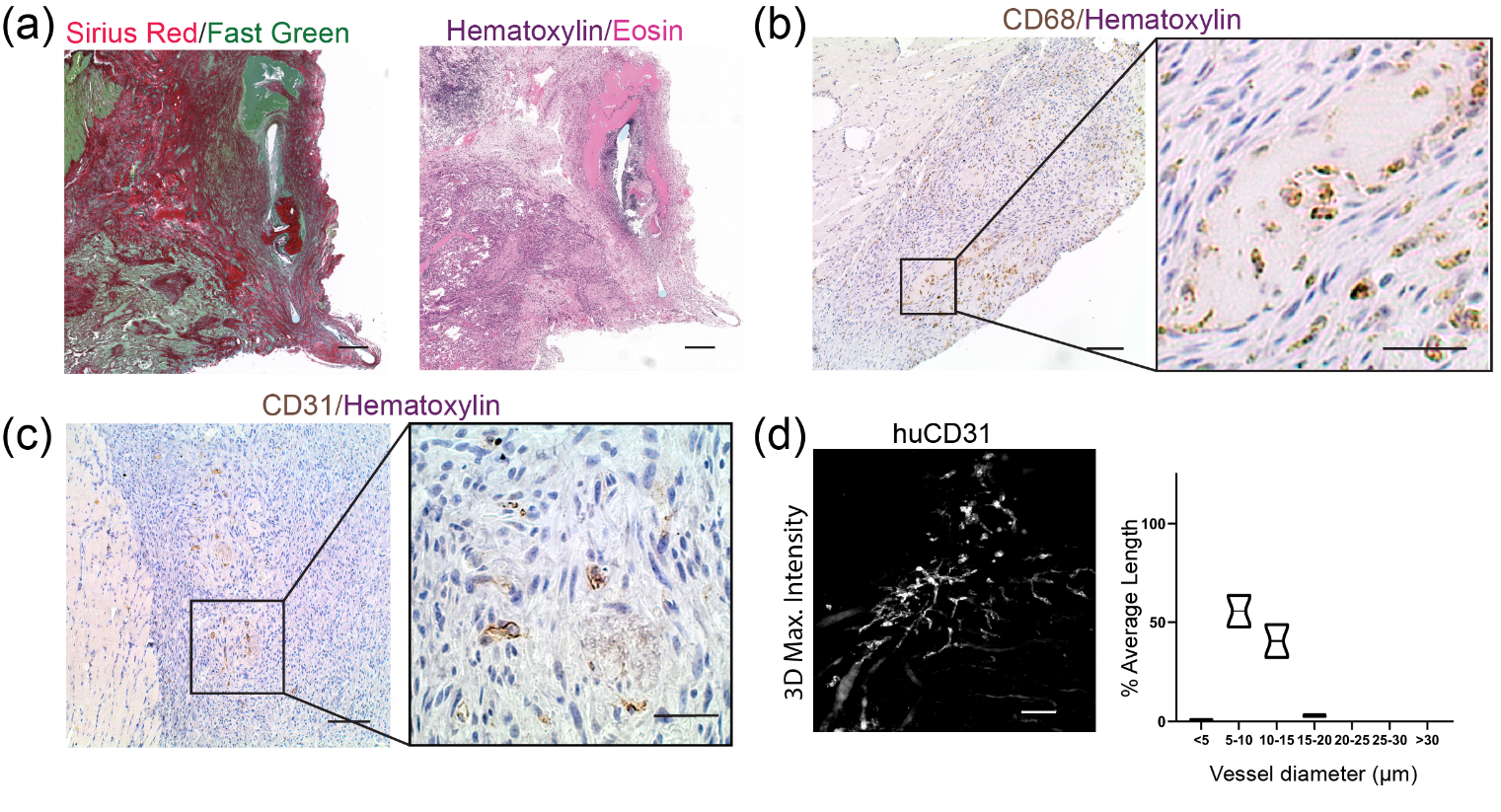
**

**Fig. S4** Fibrin patches with cords induce inflammation on the athymic rat heart. (a) Sirius Red staining reveals the fibrin matrix has been replaced by dense collagen (left). Hematoxylin & Eosin shows robust nuclear infiltration throughout the patch, with no evidence of patterned vessels (right). Scale bar = 100μm. (b) Immunostaining identifies numerous CD68+ macrophages clustered around the remaining fibrin (inset). Scale bar = 50μm, inset scale = 20μm. (c) Staining for huCD31 reveals sparse graft-derived vessels. Inset shows close view of a cord with occasional graft-derived vessels in the vicinity. Scale bar = 50μm, inset scale = 20μm (d) 3D projection of huCD31 staining from a cleared tissue (left). 3D stacks were used to generate vessel traces and quantify lumen size using Vesselucida software (right). Scale bar = 100μm.


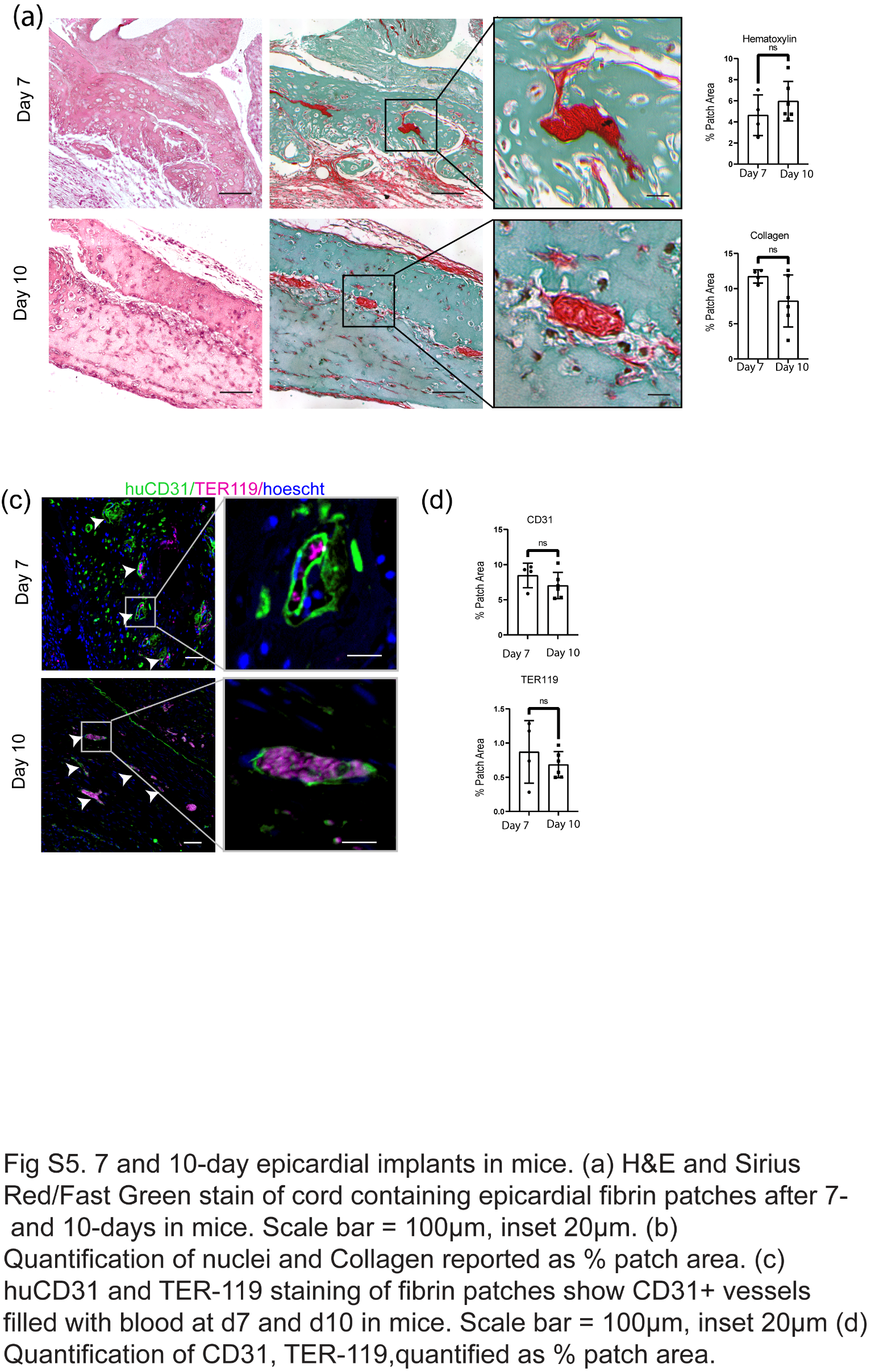


**Fig S5.** 7 and 10-day epicardial implants in mice. (a) H&E and Sirius Red/Fast Green stain of cord containing epicardial fibrin patches after 7 (n=4) - and 10-days (n=6) in mice. Scale bar = 100μm, inset 20μm. (b) Quantification of nuclei and Collagen reported as % patch area. (c) huCD31 and TER-119 staining of fibrin patches show CD31+ vessels filled with blood at d7 and d10 in mice. Scale bar = 100μm, inset 20μm (d) Quantification of CD31, TER-119, quantified as % patch area.


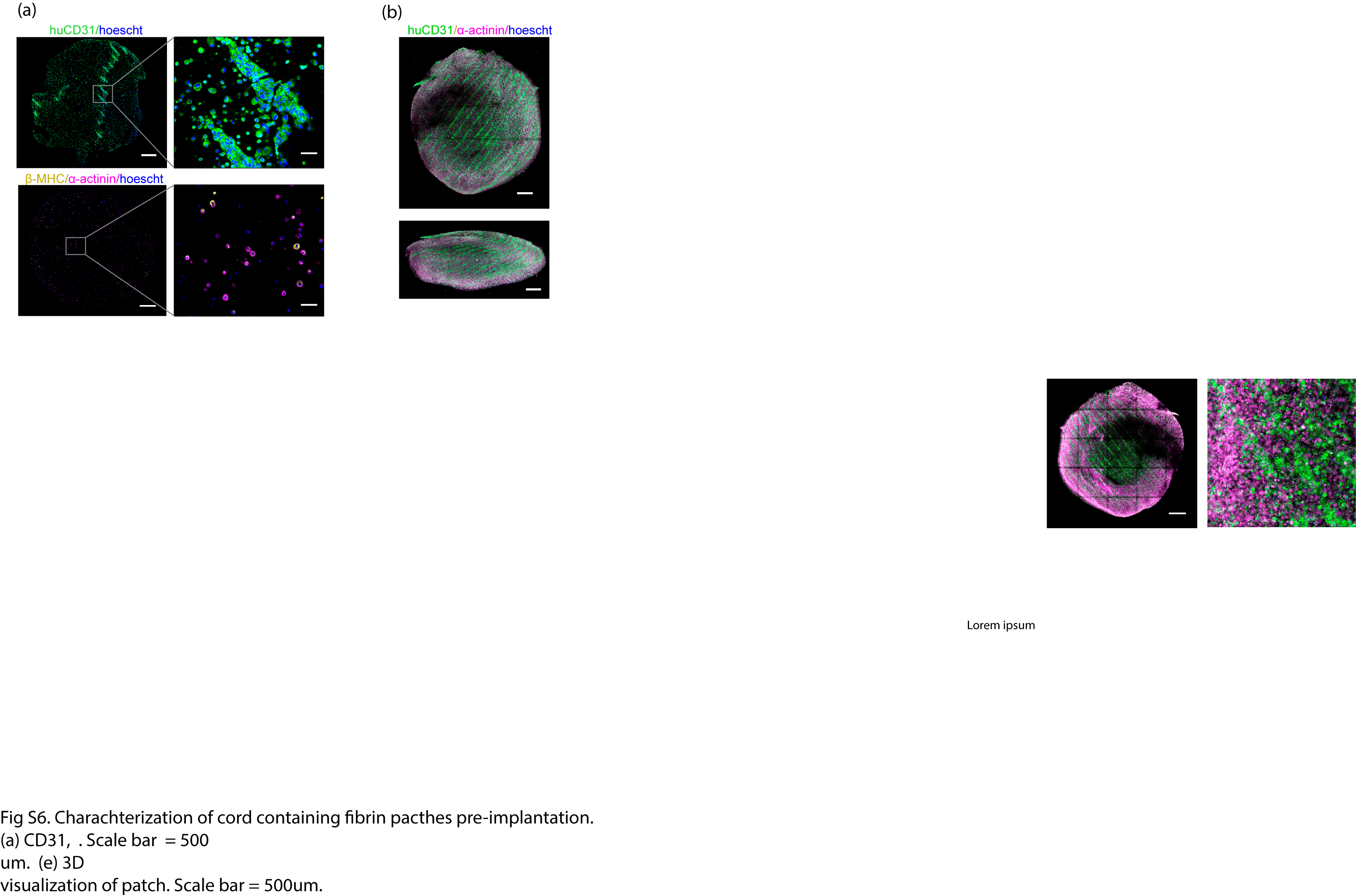


**Fig S6.** In vitro characterization of cord containing fibrin patches prior to implantation. (a) CD31 and Hoechst staining reveals patterned cords in fibrin patch pre-implantation. βMHC and α-actinin staining shows cardiomyocytes in a fibrin patch pre-implantation, scale bar = 500µm. (b) 3D visualization of an optically cleared fibrin patch stained with CD3, α-actinin, and Hoechst. Scale bar = 500µm.

**
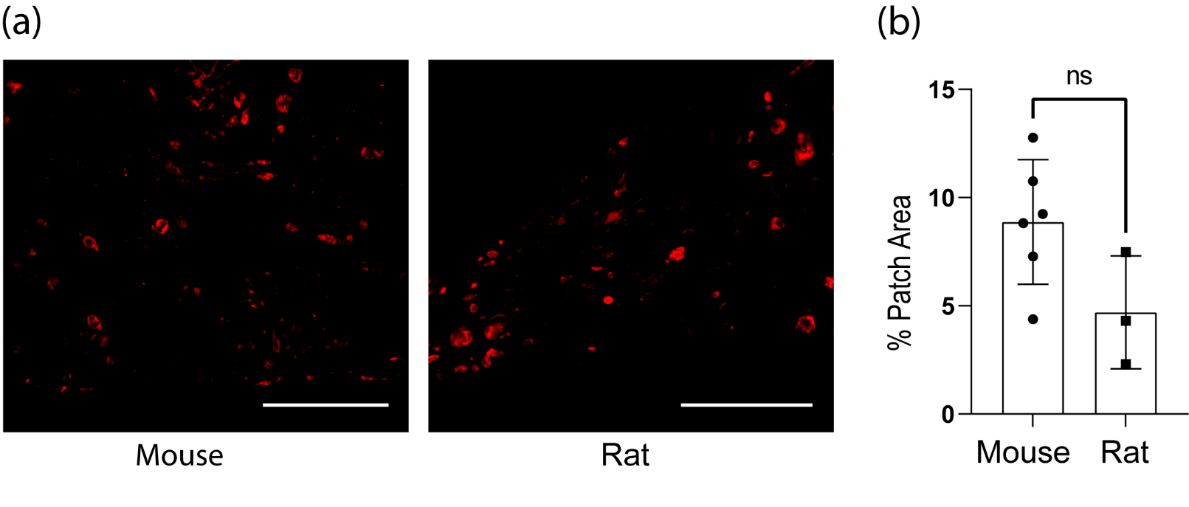
**

**Fig S7.** Mouse and Rat patches stained for rodent specific lectins. (a) 10-day mouse (n=6) and rat (n=3) patches stained with rodent specific lectins. Scale bar = 500µm. (b) Quantification of rodent specific lectin as % patch area.

**Supplementary Video 1.** Three-dimensional demonstration of CD31+ vessels from “Cords and bulk” condition patches with Vesselucida tracings. Volume = 250μm^3^

**Supplementary Video 2.** Three-dimensional demonstration of CD31+ vessels from “Cords” condition patches with Vesselucida tracings. Volume = 250μm^3^

**Supplementary Video 3.** Three-dimensional demonstration of CD31+ vessels from “bulk” condition patches with Vesselucida tracings. Volume = 250μm^3^

**Supplementary Video 4.** Three-dimensional demonstration of patch geometry pre-implantation stained with CD31(green), α-actinin(magenta), and Hoechst (blue). Scale bar = 500 μm.
